# CD20-negative de novo diffuse large B-cell lymphoma in HIV-negative patients: A matched case-control analysis in a single institution

**DOI:** 10.1186/1479-5876-10-84

**Published:** 2012-05-03

**Authors:** Ya-Jun Li, Zhi-Ming Li, Hui-Lan Rao, Yi Xia, Hui-Qiang Huang, Zhong-Jun Xia, Su Li, Wen-Yu Li, Wen-Qi Jiang

**Affiliations:** 1 State Key Laboratory of Oncology in Southern China, Guangzhou, 510060, China; 2 Department of Medical Oncology, Sun Yat-Sen University Cancer Center, Guangzhou, 510060, China; 3 Department of Pathology, Sun Yat-Sen University Cancer Center, Guangzhou, 510060, China; 4 Department of Hematological Oncology, Sun Yat-Sen University Cancer Center, Guangzhou, 510060, China; 5 Division of Lymphoma, Cancer Center, Guangdong General Hospital, Guangzhou, 510089, China

**Keywords:** Diffuse large B-cell lymphoma, CD20-negative, Clinicopathologic features, HIV-negative

## Abstract

**Background:**

HIV-negative, CD20-negative *de novo* diffuse large B-cell lymphoma (DLBCL) patients has rarely been reported. To elucidate the nature of this entity, we retrospectively reviewed the data of 1,456 consecutive *de novo* DLBCL patients who were treated at Sun Yat-Sen University Cancer Center between January 1999 and March 2011.

**Methods:**

The pathologic characteristics of CD20-negative patients, clinical features, response to initial treatment, and outcomes of 28 patients with available clinical data (n = 21) were reviewed. Then, a matched case-control (1:3) analysis was performed to compare patients with CD20-negative and -positive DLBCL.

**Results:**

The median age of the 28 CD20-negative DLBCL patients was 48 years, with a male-female ratio of 20:8. Seventeen of 22 (77.3%) CD20-negative DLBCL cases were of the non-germinal centre B-cell (non-GCB) subtype. High Ki67 expression (≥80%), an index of cell proliferation, was demonstrated in 17 of 24 (70.8%) cases. Extranodal involvement (≥ 1 site) was observed in 76.2% of the patients. Following initial therapy, 9 of 21 (42.9%) cases achieved complete remission, 4 (19%) achieved partial remission, 1 (4.8%) had stable disease, and 7 (33.3%) had disease progression. The median overall survival was 23 months. The 3-year progression-free survival (PFS) and overall survival (OS) rates were 30.5% and 35%, respectively. A matched case-control analysis showed that patients with CD20-negative and -positive DLBCL did not exhibit a statistically significant difference with respect to the main clinical characteristics (except extranodal involvement), whereas the patients with CD20-positive DLBCL had a better survival outcome with 3-year PFS (*P* = 0.008) and OS (*P* = 0.008) rates of 52% and 74.1%, respectively.

**Conclusions:**

This study suggests that HIV-negative, CD20-negative *de novo* DLBCL patients have a higher proportion of non-GCB subtype, a higher proliferation index, more frequent extranodal involvement, a poorer response, and a poorer prognosis to conventional treatment compared to patients with CD20-positive DLBCL. Further studies are warranted to investigate new target and optimal therapy of CD20-negative *de novo* DLBCL.

## Background

Diffuse large B-cell lymphoma (DLBCL) is the most frequent subtype of non-Hodgkin’s lymphoma (NHL) in Western and Eastern countries, representing 30%-40% of all non-Hodgkin’s lymphoma cases [1,2]. CD20 antigen is a membrane-bound protein which plays a role in B-cell activation, differentiation, and cell-cycle progression [3-5]. CD20 is an excellent pan-B-cell immunophenotypic marker because CD20 is highly expressed on the surface of 90%-95% of normal and neoplastic B lymphocytes; CD20 is not expressed on immature B precursors and plasma cells [6,7]. Recent studies have demonstrated that CD20-negative DLBCL is frequently restricted to a few variant subtypes of DLBCL with plasmablastic features and terminal B-cell differentiation, including plasmablastic lymphoma (PBL) of the oral mucosa type, PBL with plasmacytic differentiation, primary effusion lymphoma (PEL), Kaposi’s sarcoma-associated herpes virus (KSHV)-positive solid lymphoma/extracavitary PEL/HHV-8 associated DLBCL, and ALK-positive DLBCL [8-14]. Limited studies with a large series of cases have focused on the clinical and pathologic features of HIV-negative, CD20-negative *de novo* DLBCL patients [9-14].

To shed light on the nature of the entity, we conducted a matched case-control analysis to compare the clinicopathologic characteristics and clinical outcome of HIV-negative, CD20-negative DLBCL and -positive DLBCL patients at a single institution. This is the first matched case-control analysis to investigate HIV-negative, CD20-negative DLBCL patients.

## Materials and methods

### Patient selection

We retrospectively reviewed the pathologic data of 1,456 consecutive patients with *de novo* DLBCL diagnosed by experienced hematopathologists at Sun Yat-Sen University Cancer Center between January 1999 and March 2011. Twenty-eight patients were diagnosed as CD20-negative DLBCL. Among these 28 patients, 21 patients received treatment at Sun Yat-Sen University Cancer Center and had available clinical information and follow-up data.

### HIV infection test

All 21 patients with CD20-negative DLBCL were initially screened for HIV infection status before initial therapy. Blood samples were collected and tested for antibodies to HIV-1 and -2 using a chromatographic qualitative enzyme-linked immunosorbent assay (ELISA) test kit (Atlas Link Biotech Co., Ltd., USA) according to the manufacturer’s instruction. If samples were positive by initial ELISA, the results were further confirmed by Western blot.

### Histologic review

All hematoxylin-eosin (HE) paraffin sections of the 28 patients were retrospectively reviewed by at least two pathologists at Sun Yat-Sen University Cancer Center to confirm the pathologic diagnosis of DLBCL according to the criteria of the 2008 World Health Organization classification [15].

### Immunohistochemical studies

Immunohistochemical (IHC) analysis was performed using a large pane of monoclonal and polyclonal antibodies detecting CD20 (L26, 1:200), CD79a (1:50), CD45 (LCA,1:20), CD3 (1:200), CD5 (1:100), CD10 (1:50), BCL-6 (1:10), MUM-1 (1:50), BCL-2 (1:80), Ki-67 (1:100), CD30 (1:20), CD38 (1:10), CD138 (1:50), UCHL-1 (CD45RO,1:200), κ (1:300), λ (1:400), OCT-2 (1:500), BOB-1 (1:500), CYCLIN D1 (1:50), ALK (1:10), CD43 (1:320), PAX-5, and Vs38c (P63,1:10) antigens (Dako, Glostrup, Denmark). Sections (4 μm thick) were cut from each paraffin block, deparaffinized, and incubated at 121^o^C in citrate buffer (pH 6.0) for 10 min for antigen retrieval. A routine immunohistochemistry method was performed for immunostaining the above antigens, as described previously [16]. For semi-quantitative evaluation of immunostained sections, we used a cut-off value of 10% to determine CD20 expression or absence according to the following criteria: CD20-negative (immunostaining of 0-10% of tumor cells); and CD20-positive (immunostaining of > 10% of tumor cells). The cut-off value we used in the present study for other proteins (except Ki-67) was 30%, according to the following previously published criteria: negative expression (immunostaining of 0-30% of tumor cells); and positive expression (immunostaining of > 30% of tumor cells) [17]. A Ki-67 ≥ 80% was consistent with a high proliferation index of lymphoma cells. In all cases, the percentage of immunostained tumor cells was consensually estimated by at least two pathologists on a multi-headed microscope. To make sure the results were as reliable as possible, loss of CD20 expression was confirmed by at least two experienced hematopathologists in our center. Furthermore, the samples without CD20 expression were re-immunostained for CD20 to confirm the findings. Patients were sub-classified into germinal centre B-cell-like (GCB) and non-GCB groups based on the algorithm of Hans et al. [17].

### *In situ* hybridization and fluorescence *in situ* hybridization study

*In situ* hybridization (ISH) analysis for Epstein-Barr virus (EBV)-encoded small RNAs (EBERs) was performed on paraffin sections of lymphoma tissues with fluorescein-conjuated peptide nucleic acid probes (Dako), according to the manufacturer’s instructions. Fluorescent *in situ* hybridization (FISH) analysis was performed to detect the translocation of chromosomes in select cases.

### Matched case-control study design

Patients who did not receive rituximab treatment during the course of their disease were selected from the remaining 1,428 CD20-positive patients with *de novo* DLBCL diagnosed at Sun Yat-Sen University Cancer Center between January 1999 and March 2011 to serve as matched controls of patients with CD20-negative DLBCL. Three control cases were matched to each study patient with CD20-negative DLBCL. For the 21 CD20-negative DLBCL with both pathologic and clinical data, the matching criteria was as follows: a standard international prognosis index (IPI) score (age, Eastern Cooperative Oncology Group PS, lactate dehydrogenase [LDH] level, Ann Arbor stage, and number of extranodal sites); gender (male or female); and age (±5 years). All of the above three factors were fully matched between the study case and the three controls. For the remaining 7 CD20-neagtive DLBCL cases with only pathologic data, the matching criteria was as follows: gender (male or female); and age (the same age). Both factors were matched between the case and the three controls. If greater than three controls were matched with a case, three were picked randomly.

This study was approved by the Institutional Review Board of the National Cancer Institute, as well as the Ethics Committee of Sun Yat-Sen University Cancer Center. The study was conducted in accordance with the Declaration of Helsinki and the institutional guidelines of the local Ethics Committee.

### Statistical methods

Progression-free survival (PFS) was defined as the interval between the date of diagnosis and the date of first relapse, progression, death, or last follow-up. Overall survival (OS) was defined from the day of diagnosis until the time of death or last follow-up. The survival curve was constructed by the Kaplan-Meier method, and comparisons between groups were made using the log-rank test. The following clinicopathologic variables associated with survival in common DLBCL were dichotomized to facilitate univariate analysis for survival of HIV-negative, CD20-negative *de novo* DLBCL patients, which were compared using the Kaplan-Meier method and log-rank test: age (>60 years vs. ≤ 60 years), Eastern Cooperative Oncology Group (ECOG) performance status (PS; >1 vs. ≤ 1), stage (stage I/IIvs. stage III/IV), bulky (≥ 7 cm vs. < 7 cm), serum LDH (normal vs. elevated), extranodal involvement (≥ 2 vs. < 2), IPI (0-1 vs. 2-3), Ki-67 (≥ 80% vs. <80%), BCL-2 (positive vs. negative), molecular subtypes (GCB vs. non-GCB), response to initial therapy (complete remission [CR]/partial remission [PR] vs. stable disease [SD]/progressive disease [PD]).

The clinicopathologic variables of the two groups were compared using a *χ*^2^ test for categorical variables and the Mann-Whitney test for continuous variables. A two-tailed *P*-value <0.05 was considered to be statistically significant. The statistical software package, SPSS 16.0 (SPSS, Inc., Chicago, IL, USA), was used for statistical calculations.

## Results

### CD20-negative DLBCL patients

#### Clinical features and HIV test results

For all 28 cases, the median age was 48 years (range, 11-83 years) and the male-to-female ratio was 20:8. Twenty-one cases had complete clinical data and follow-up information. The baseline clinical information is summarized in Tables 1 and 2. All of the 21 cases were HIV-negative and had no history of other lymphoproliferative disorders, organ/hematopoietic stem cell transplantation, or congenital immunodeficiencies. Twelve cases (57.1%) had stage I/II disease, and 9 patients (42.9%) had stage III/IV disease according to the Ann Arbor staging system. B symptoms were present in 8 cases (38.1%). Bulky disease (mass ≥ 7 cm) was observed in 8 cases (38.1%). The serum lactate dehydrogenase (LDH) level was elevated in 7 cases (33.3%). Sixteen patients (76.2%) had extranodal involvement (11 cases with 1 site, and 5 cases with > 1 site). Eleven patients (52.4%) had low International Prognostic Index (IPI) scores (0-1 risk factor), 5 patients (23.8%) had low-intermediate IPI scores (2 risk factors), the IPI score was 3 (3 risk factors) in 5 patients (23.8%), and there were no patients with high-risk IPI scores (4-5 risk factors). The bone marrow was involved in 2 patients (9.5%). The PS was < 2 in 18 patients (85.7%).

**Table 1:** Clinical, treatment and survival characteristics of the 21 patients with CD20-negative diffuse large B-cell lymphoma

**Patient NO.**	**Age, y/Sex**	**HIV status**	**PS score**	**Site of involvement**	**Stage**	**Bulky disease**	**Serum LDH**	**IPI**	**Therapy and response for initial therapy**	**Out come**	**Survival (months)**
1	68/M	-	1	LN	IIB	No	Normal	1	CHOP × 6+RT, ICE × 4,CR	AWD	117
2	33/F	-	1	Cervical	IA	No	Normal	0	CHOP × 4, CR	AND	117
3	49/F	-	1	Maxillary sinus, LN	IIB	No	Normal	0	CHOP × 5, DHAP × 4, CR	DOD	11
4	11/M	-	2	Spinal canal, Retroperitoneal	IIIB	Yes	Normal	3	Surgical resection+RT, CAV × 2 + BFM-90 × 1, PD	DOD	23
5	37/M	-	1	Retroperitoneal, LN, BM	IVB	Yes	Elevated	2	CHOP × 2,VAD × 2,MEA × 2, PR	AND	90
6	56/M	-	1	Stomach, Retroperitoneal,	IIB	No	Normal	0	CHOP × 6,ICE × 2,DHAP × 3, GEMOX × 7,Auto PBSCT, PR	DOD	33
7	54/F	-	1	Stomach	IIB	Yes	Normal	0	Gastrectomy, CHOP × 6,ICE × 2, EPOCH × 1,FC × 1, PD	DOD	13
8	73/M	-	1	LN	IA	No	Elevated	2	CHOP × 2, PD	DOD	7
9	44/M	-	1	LN	IIIA	No	Elevated	2	CHOP × 6,HD-MTX +Ara-C × 1, PD	DOD	6
10	41/M	-	2	Stomach, LN	IIA	No	Elevated	1	CHOP × 2, PD	DOD	3
11	66/M	-	1	Stomach, LN	IIB	Yes	Elevated	2	EPOCH × 6+RT, CR	AND	27
12	22/M	-	1	Liver, lung, LN, hip	IVB	No	Elevated	3	CHOP × 1,DHAP × 2, IMVP-16 × 4, PD	DOD	13
13	55/M	-	1	LN	IA	No	Normal	0	Bortezomib+EPOCH × 4, RT, CR	AND	25
14	19/F	-	1	Stomach, LN	IIA	Yes	Normal	0	Gastrectomy, CHOP × 6, CR	AND	20
15	67/M	-	1	Spleen, LN	IIIA	No	Elevated	3	CHOP × 1, ICE × 4,SD	AWD	19
16	47/F	-	1	Ileum, liver, LN,	IVA	Yes	Normal	2	Surgical resection, CHOP × 1, MAID × 2,GEMOX × 4,ESHAP × 2, PD	DOD	12
17	27/M	-	1	Mediastinum, pleural	IIA	Yes	Normal	0	Surgical resection, CHOP+IT × 6, CR	AND	15
18	83/M	-	1	Lung, rib, LN	IVA	No	Normal	3	CHOP × 3, GEMOX × 2, CR	DOD	7
19	53/M	-	1	Stomach, LN	IIIA	No	Normal	1	CHOPE × 6,GIFOX × 2,GND × 2, CR	AND	9
20	67/M	-	1	LN	IA	No	Normal	1	RT,CHOP × 2,Ara-C+DXM × 1, Ara-C+Paclitaxel × 1, PR	AWD	9
21	71/M	-	2	Kidney, sternum, vertebral, BM	IVA	Yes	Normal	3	CHOP × 2, ICE × 2, PR	DOD	7

Note: -, negative; +, positive; PS, performance status; LN, lymph node; BM, bone marrow; LDH, lactate dehydrogenase; IPI, International Prognostic Index; RT, radiotherapy; CAV, cyclophosphamide, doxorubicin, vincristine; VAD, vincristine, doxorubicin, dexamethasone; MEA, mitoxantrone, etoposide, cytarabine; GEMOX, gemcitabine, oxaliplatin; GIFOX, gemcitabine, ifosfamide, oxaliplatin; GND, gemcitabine, vinorelbine, doxil; DXM, dexamethasone; MAID, mesna, doxorubicin, ifosfamide, dacarbazine; PBSCT, peripheral blood stem cell transplant; IT, intrathecal; CR, complete remission; PR, partial remission; SD, stable disease; PD, progressive disease; AWD, alive with disease; AND, alive with no evidence of disease; DOD, died of disease.

**Table 2:** Clinical characteristics of CD20-negative diffuse large B-cell lymphoma and CD20-positive diffuse large B-cell lymphoma patients

	**Total**	**CD20-negative DLBCL(n = 21)**	**CD20-positive DLBCL (n = 63)**	***P***
**Age(years)**				
Median (range)	51.5(9-83)	53(11-83)	51(9-71)	0.955
≤60	56(66.7%)	14(66.7%)	42(66.7)	1
**Sex**				
Male	64(76.2%)	16(76.2%)	48(76.2%)	1
Female	20(23.8%)	5(23.8%)	15(23.8%)	
**PS score**				
0-1	75(89.3%)	18(85.7%)	57(90.5%)	0.839
≥2	9(10.7%)	3(14.3%)	6(9.5%)	
**Stage**				
I-II	49(58.3%)	12(57.1%)	37(58.7%)	0.898
III-IV	35(41.7%)	9(42.9%)	26(41.3%)	
**LDH**				
Normal	50(59.5%)	14(66.7%)	36(57.1%)	0.441
Elevated	34(40.5%)	7(33.3%)	27(42.9%)	
**Extranodal sites**				
0-1	73(86.9%)	16(76.2%)	57(90.5%)	0.191
≥2	11(13.1%)	5(23.8%)	6(9.5%)	
**IPI**				
0-1	44(52.4%)	11(52.4%)	33(52.4%)	1
2-3	40(47.6%)	10(47.6%)	30(47.6%)	
**Bulky disease**				
Yes	20(23.8%)	8(38.1%)	12(19%)	0.076
No	64(76.2%)	13(61.9%)	51(81%)	
**BM involvement**				
Yes	2(2.4%)	2(9.5%)	0(0)	0.06
No	82(97.6%)	19(90.5%)	63(100%)	
**B symptom**				
Present	23(27.4%)	8(38.1%)	15(23.8%)	0.204
Absent	61(72.6%)	13(61.9%)	48(76.2%)	
**First-line chemotherapy**				
CHOP or CHOP-like	79(94%)	20(95.2%)	59(93.7%)	1
Other regimens	5(6%)	1(4.8%)	4(6.3%)	
**Radiotherapy**				
Yes	28(33.3%)	5(23.8%)	23(36.5%)	0.285
No	56(66.7%)	16(76.2%)	40(63.5%)	
**Surgery**				
Yes	11(13.1%)	5(23.8%)	6(9.5%)	0.191
No	73(86.9%)	16(76.2%)	57(90.5%)	
**Autologous SCT**				
Yes	4(4.8%)	1(4.8%)	3(4.8%)	1
No	80(95.2%)	20(95.2%)	60(95.2%)	
**Follow-up (months)**				
Median (range)	41.5(2-121)	13(3-117)	47(2-121)	

PS, performance status; LDH, lactate dehydrogenase; IPI, international prognosis index; BM, bone marrow; SCT, stem cell transplantation.

#### Histologic and immunohistochemical features, and ISH and FISH studies

**Figure 1:**
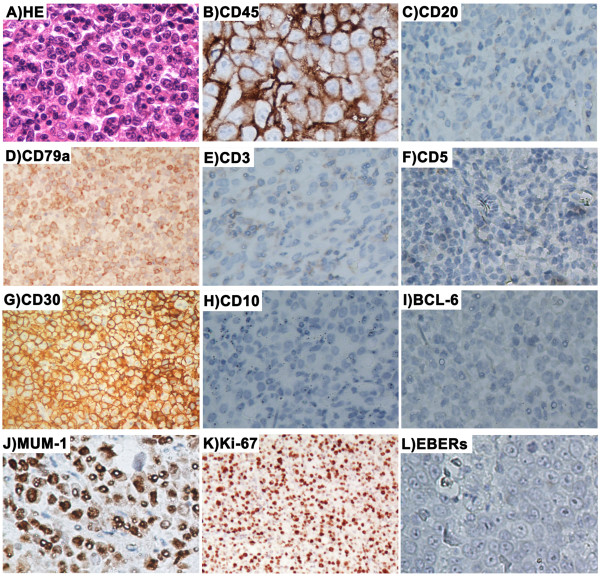
**Typical morphology and immunophenotype of CD20-negative diffuse large B-cell lymphoma ranged from A to L.** A (Hematoxylin-Eosin staining): Diffuse proliferation of large tumor cells with large nucleus, prominent nucleoli, abundant cytoplasm, and the karyokinesis were easy to be observed; B: Tumor cells were positive for CD45 (LCA); C: Tumor cells were negative for CD20; D: Tumor cells were positive for CD79a; E-F: Tumor cells were negative for CD3, CD5; G: Tumor cells were strong positive for CD30; H-I: Tumor cells were negative for CD10 and BCL-6; J: Tumor cells were positive for MUM-1; K: Tumor cells had a very high proliferation index with Ki-67 highlighting more than 90% tumor cells; L: Tumor cells were negative for EBERs ISH.

We reviewed the pathologic data of 1,456 consecutive patients with *de novo* DLBCL diagnosed at Sun Yat-Sen University Cancer Center between January 1999 and March 2011. Twenty-eight cases (1.9%) met the criteria for CD20-negative DLBCL. Based on the IHC results and microscopic morphologic characteristics, 2 of 28 patients were diagnosed as PBL, 3 patients were diagnosed as DLBCL with plasmacytic differentiation, and 5 patients were diagnosed as ALK-positive DLBCL according to the WHO criteria. There were no patients diagnosed as PEL among the 28 cases. The microscopic morphologic characteristics of 5 ALK-positive DLBCL patients and 2 PBL patients were as follows: ALK-positive DLBCL, the tumor showed a sinusoidal growth pattern and was composed of monomorphic large immunoblast-like cells with round pale nuclei containing large central nuclei and abundant cytoplasm, with multinucleated neoplastic giant cells in 3 cases; PBL, the tumor showed a diffuse and cohesive proliferation of cells resembling immunoblasts and mitotic figures were observed in tumor cells. For the remaining 21 conventional DLBCL cases, typical morphology of the lymphoma cells was characterized by large lymphoid cells diffusely infiltrated lymph nodes or other tissues. Most of the cells were centroblasts and cells with plasmacytic differentiation were noted in three cases. Centroblasts were medium-to-large lymphoid cells with oval-to-round, vesicular nuclei containing fine chromatin. There were two-to-four nuclear membrane-bound nucleoli. The cytoplasm was usually scanty and amphophilic-to-basophilic (Figure 1A).

The major immunophenotypic features are summarized in Tables 3 and 4. All 28 patients were negative for CD20. The immunophenotypes are listed as follows (+/total): CD45, 26 of 27 (96.3%); CD79a, 23 of 26 (88.5%); BOB-1, 9 of 11 (81.8%); OCT-2, 9 of 12 (75%); Pax-5, 5 of 11 (45.5%); CD10, 5 of 22 (22.7%); BCL-6, 2 of 15 (13.3%); MUM-1, 16 of 21 (76.2%); BCL-2, 13 of 20 (65%); CD30, 9 of 25 (36%); CD38, 8 of 22 (36.4%); CD138, 5 of 22 (22.7%); kappa, 5 of 11 (45.5%); lambda, 5 of 10 (50%); ALK, 5 of 24 (20.8%); CD43, 6 of 14 (42.9%); VS38c (P63), 6 of 15 (40%); CYCLIN D1, 0 of 12; CD5, 0 of 23; and CD23, 0 of 11. Of the T cell- or NK/T cell-associated markers (CD3 and CD56, respectively), 0 of 26 and 0 of 9 were positive in lymphoma cells, respectively. Interestingly, the T cell-associated antigen, UCHL-1(CD45RO), was positive in 3 of 23 (13%) cases. According to the Hans algorithm [17], 17 of 22 (77.3%) cases were categorized as non-GCB type, while 5 of 22 (22.7%) cases were categorized as GCB type. Ki-67 was immunolabelled as a high proliferation index (≥ 80%) for lymphoma cells in 17 of 24 (70.8%) cases (Figure 1K).

**Table 3:** The immunohistochemical features and EBV status of 28 patients with CD20-neagtive diffuse large B-cell lymphoma

**Patient NO.**	**Age, y/Sex**	**Sampling Site**	**CD45**	**CD20**	**CD79a**	**CD5**	**CD3**	**BCL-2**	**CD10**	**BCL-6**	**Mum-1**	**Ki-67 (%)**	**EBER ISH**
1	68/M	LN	+	-	+	-	-	-	-	NT	+	90	-
2	33/F	Cevical	+	-	NT	NT	NT	NT	NT	NT	NT	NT	NT
3	49/F	Maxillary Sinus	+	-^†^	+	NT	-	NT	NT	NT	NT	NT	NT
4	11/M	Spinal canal	+	-	+	-	-	+	-	-	+	80	-
5	37/M	Retroperitoneal	+	-	+	-	-	-	NT	NT	NT	80	-
6	56/M	Retroperitoneal	+	-	+	-	-	+	-	-	NT	90	-
7	54/F	Stomach	+	-	+	-	-	-	-	-	+	80	-
8	73/M	LN	+	-	+	-	-	-	-	-	+	80	+
9	44/M	LN	+	-	+	-	-	NT	-	-	+	90	NT
10	41/M	Stomach	+	-	+	NT	-	NT	NT	NT	NT	NT	NT
11	66/M	Stomach	+	-	+	-	-	NT	-	-	-	80	-
12	22/M	Hip	+	-	+	NT	-	NT	NT	NT	-	NT	NT
13	55/M	LN	NT	-	-	-	-	NT	-	NT	+	70	-
14	19/F	Stomach	+	-	+	-	-	+	-	NT	+	90	-
15	67/M	LN	+	- ^‡^	+	-	-	-	-	-	+	70	-
16	47/F	Liver	+	-	+	-	-	+	-	-	+	90	-
17	27/M	Pleural	+	-	+	-	NT	+	-	-	+	60	-
18	83/M	LN	+	-	+	-	-	+	+	+	+	100	NT
19	53/M	Stomach	+	-^§^	+	-	-	+	+	NT	+	100	-
20	67/M	LN	+	-^¶^	+	-	-	+	-	+	+	90	-
21	71/M	Sternum	+	-	+	-	-	NT	+	NT	NT	90	-
22	62/F	LN	+	-	+	-	-	+	+	NT	+	80	-
23	64/M	Maxillary sinus	+	-	NT	NT	-	+	-	-	+	80	NT
24	35/M	Lung	+	-	-	-	-	-	+	NT	-	60	-
25	40/F	Cevical	-	-	-	-	-	+	-	-	-	30	-
26	18/M	LN	+	-	+	-	-	+	NT	NT	NT	30	-
27	24/F	Ovarian	+	-	+	-	-	-	-	-	-	70	-
28	27/M	Mediastinum	+	-	+	-	-	+	-	-	+	100	-

Note: LN, lymphoma node; -, negative; +, positive; NT, not tested; †, ‡, §, ¶: CD20 immunostaining in patient 3, 15, 19 and 20 were performed 2, 3, 4 and 2 times, respectively. All the results of CD20 immunostaining were negative.

**Table 4:** Main pathological characteristics of CD20-negative diffuse large B-cell lymphoma and CD20-positive diffuse large B-cell lymphoma patients

	**Total (tested)**	**CD20-negative DLBCL(n = 28)**	**CD20-positive DLBCL(n = 84)**	***P***
**IHC Subtypes**				
GCB	26(48.1%)	5(22.7%)	21(65.6%)	0.002
non-GCB	28(51.9%)	17(77.3%)	11(34.4%)	
**Ki-67**				
≥80%	30(42.3%)	17(70.8%)	13(27.7%)	<0.001
<80%	41(57.7%)	7(29.2%)	34(72.3%)	
**BCL-2**				
Positive	37(54.4%)	13(65%)	24(50%)	0.258
Negative	31(45.6%)	7(35%)	24(50%)	
**EBERs**				
Positive	2(6.9%)	1(4.8%)	1(12.5%)	0.483
Negative	27(93.1%)	20(95.2%)	7(87.5%)	
**CD30**				
Positive	14(19.4%)	9(36%)	5(10.6%)	0.023
Negative	58(80.6%)	16(64%)	42(89.4%)	
**ALK**				
Positive	7(20.6%)	5(20.8%)	2(20%)	1
Negative	27(79.4%)	19(79.2%)	8(80%)	
**Specified DLBCL**				
ALK-positive LBCL	5(4.5%)	5(17.9%)	0(0)	0.001
PBL	2(1.8%)	2(7.1%)	0(0)	0.061
PEL	0(0)	0(0)	0(0)	1
**DLBCL with PD**	4(3.6%)	3(10.7%)	1(1.2%)	0.078

IHC, Immunohistochemical; GCB, germinal centre B-cell; DLBCL, diffuse large B-cell lymphoma; LBCL, large B-cell lymphoma; PBL, plasmablastic lymphoma; PEL, primary effusion lymphoma; PD, plasmacytic differentiation.

Only 1 of 21 cases (4.8%) tested for EBV infection had positive signals in the nuclei of the lymphoma cells in EBERs ISH. Both IgH-MALT-1/t (14; 18) and API-2-MALT-1/t (11; 18) were negative in the one case tested by FISH.

#### Response and survival analysis

**Figure 2:**
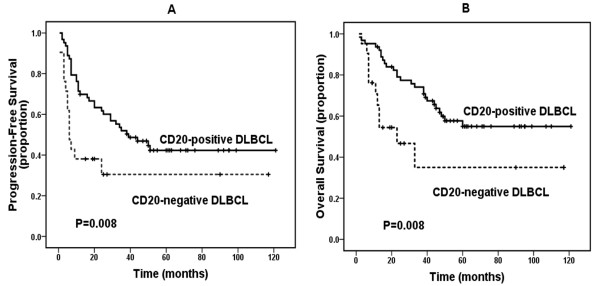
**Progression-free survival (PFS) and overall survival (OS) of patients with CD20-negative diffuse large B-cell lymphoma (DLBCL) and CD20-positive DLBCL.** (A): PFS of patients with CD20-negative DLBCL (n = 21, dotted line) and patients with CD20-positive DLBCL (n = 63, solid line). (B): OS of patients with CD20-negative DLBCL (n = 21, dotted line) and patients with CD20-positive DLBCL (n = 63, solid line).

Twenty of 21 CD20-negative *de novo* DLBCL cases received cyclophosphamide, doxorubicin, vincristine, and prednisone (CHOP) or a CHOP-like regimen chemotherapy as first-line chemotherapy. Five cases received radiotherapy pre- or post-chemotherapy. Five patients underwent surgical resection as initial treatment. Only one patient received autologous stem cell transplantation (SCT) during the course of the disease. Following initial therapy, 9 of 21 (42.9%) cases achieved CR, 4 (19%) achieved PR, 1 (4.8%) had SD, and 7 (33.3%) had PD. By the time of analysis, 11 patients (38.1%) had died; all of the deaths were due to lymphoma. The median OS time was 23 months. The estimated 3-year PFS and OS rates were 30.5% and 35%, respectively (Table 5 and Figures 2A, B). Based on univariate analysis, 3 variables associated with a longer OS included a PS ≤ 1 (*P* = 0.033), extranodal involvement < 2 sites (*P* = 0.027), and a CR/PR response to initial therapy (*P* = 0.008); however, age > 60 years, advanced stage (stage III/IV), IPI score (≥ 2), bulky disease (≥ 7 cm), elevated LDH, high Ki-67, non-GCB subtype, and positive BCL-2 were not associated with inferior survival (all *P*>0.05).

**Table 5:** Response to first-line chemotherapy and survival in patients with CD20-negative diffuse large B-cell lymphoma and CD20-positive diffuse large B-cell lymphoma

	**CD20-negative DLBCL(n = 21)**	**CD20-positive DLBCL (n = 63)**	***P***
**Response**			
Assessable, n	21	63	
CR	9 (42.9%)	52(82.5%)	0.001
No CR	12(57.1%)	11(17.5%)	
**Survival**			
3-years PFS	30.5%	52%	0.008
Median PFS, months (range)	6(1-117)	39(2-121)	
3-years OS	35%	74.1 %	0.008
Median OS, months (range)	23(3-117)	Not reached(2-121)	

DLBCL, diffuse large B-cell lymphoma; CR, complete remission; PFS, progression-free survival; OS, overall survival.

### Matched case-control analysis

#### Clinical characteristics

As expected from the matching method, the two groups of patients did not show any statistically significant difference in the main clinical characteristics (Table 2)**;** however, extranodal involvement (≥1 site) was more frequent in CD20-negative DLBCL patients (76.2% vs. 44.4%, *P* = 0.012).

#### Pathologic characteristics

The main pathologic characteristics of the two groups are listed in Table 4. Compared with CD20-positive DLBCL in the control group, the non-GCB subtype was significantly more frequent in CD20-negative DLBCL (34.4% vs. 77.3%, *P* = 0.002). Similarly, the proportion of CD20-negative DLBCL cases with Ki-67 expression ≥ 80% was significantly higher than the CD20-positive DLBCL control group (70.8% vs. 27.7%, *P*<0.001). Interestingly, CD30-positive expression was more frequent in the CD20-negative DLBCL group (36% vs. 10.6%, *P* = 0.023). There was no significant difference in the rate of other markers, such as BCL-2, EBERs, and ALK between the study and control groups. However, ALK-positive DLBCL was more frequent in the CD20-negative DLBCL group than the CD20-positive DLBCL group (17.9% vs. 0, *P* = 0.001). Furthermore, the statistical analysis showed no significant difference in rates of other specified DLBCL subtypes (PBL and PEL) and DLBCL with plasmacytic differentiation between the two groups of patients (all *P*>0.05).

#### Response to first -chemotherapy and survival

There were 95.2% and 93.7% CD20-negative DLBCL and CD20-positive DLBCL patients received CHOP or CHOP-like treatment as first-line chemotherapy, respectively. The CR rate of the control group was significantly higher than the study group (82.5% vs. 42.9%, *P* = 0.001; Table 5). With a median follow-up of 47 months (range, 2-121 months), the estimated 3-year PFS and OS rates of the control group were 52% and 74.1% (Table 5 and Figure 2), respectively. The 3-year PFS and OS in the CD20-negative DLBCL group were significantly poorer than the CD20-positive DLBCL group (*P* = 0.008 and *P* = 0.008, respectively; Table 5 and Figure 2).

## Discussion

CD20-negative DLBCL is very rare. The vast majority of reported cases mainly occur in a few variant subtypes of DLBCL, including PBL, PEL, and ALK-positive DLBCL [8-14]; however, data on CD20-negative *de novo* DLBCL patients are largely limited and limited to HIV-infected or other immunocompromised patients [8-14]. With the aim to improve the understanding of this unusual entity, we retrospectively analyzed HIV-negative patients with CD20-negative DLBCL from a large cohort (n = 1,456) and performed a matched case-control analysis to compare the clinicopathologic characteristics between CD20-negative and -positive DLBCL.

Early studies suggested that CD20 plays an important role in the control of normal B cell activation and progression through the cell cycle [3-5]. However, the exact mechanism by which CD20 functions in B cells and the role of CD20 in DLBCL remains unclear. Bubien et al. [18] reported that CD20 functions as a Ca^2+^ channel in B cell membranes. Although CD20 plays an essential role in B lymphocytes, CD20-negative DLBCL cells can still survive after loss of CD20 expression; the reason for this is not clear. Because CD20 is structurally similar to several ion channels [18], some CD20-independent channels and/or signal transduction pathways essential for the survival of CD20-negative DLBCL might exist. Further research is warranted to explore the possible transduction pathways in CD20-negative DLBCL.

CD20-negative DLBCL most frequently occurs in a few variant subtypes of DLBCL, including PBL and PEL in the HIV-infected population [8-11,13,19]. It appears that immunocompromised patients with these DLBCL subtypes are prone to loss of CD20; however, conclusions regarding the relationship between loss of CD20 expression in DLBCL and HIV infections have been inconsistent [19-21]. In a study by Hoffmann et al. [20], only 2% of HIV-positive DLBCL patients were negative for CD20. In contrast, Xicoy et al. [21] reported that 26% of HIV-positive DLBCL patients were negative for CD20, and there were no CD20-negative DLBCL cases among HIV-negative patients in their study. For PBL, Castillo et al. [19] reported that the rate of CD20-negative expression was higher in HIV-negative patients than HIV-positive patients (100% vs. 83%). The relative lower incidence of HIV infection in lymphoma patients in China compared to Western countries may be as a possible explanation for all 21 cases with CD20-negative DLBCL being HIV-negative in this series. Further studies are warranted to elucidate the precise relationship between loss of CD20 and HIV infection in DLBCL patients.

Pathologically, our results showed that CD20-negative DLBCL is more closely associated with aggressive pathologic parameters than CD20-positive DLBCL, with a higher proliferation index and a higher proportion of non-GCB type. In the previous two studies, 18% of cases with a Ki-67 ≥ 80% and 54% of cases with a Ki-67 ≥ 70% were reported in CD20-positive DLBCL [22,23]. In agreement with previous results, 27.7% of CD20-positive DLBCL patients in the present study had a high expression of Ki-67; however, in the current study, 70.8% of CD20-negative DLBCL patients had a high proliferation index (Ki-67 ≥ 80%). Furthermore, 77.3% of CD20-negative DLBCL patients were defined as the non-GCB subtype according to Hans et al. [17]. In comparison, the rate of non-GCB subtypes was only 34.4% and 58.4% in our control group and another Chinese study comprising conventional DLBCL [2], respectively. Interestingly, our study showed that 36% of CD20-negative DLBCL patients were CD30-positive. In contrast, CD30 expression occurred in 10.6% cases of our control group and 4%-17% of conventional DLBCL cases in other studies [24-26]. This could have potential therapeutic implication with the advent of SGN-35, an anti-CD30 monoclonal antibody drug conjugate with significant clinical efficacy in relapsed/refractory Hodgkin’s lymphoma and systemic anaplastic large-cell lymphoma [27-29]. Apart from these findings, there was no significant difference in the rates of expression of ALK, BCL-2, and EBERs between CD20-negative and -positive DLBCL.

Several studies have demonstrated that when treated with CHOP alone, CD20-positive DLBCL attained a CR rate of > 60% [30-33]. Similar results were observed in our CD20-positive DLBCL patients (a CR rate of 82%) when treated with CHOP or CHOP-like chemotherapy; however, our study showed that less than one-half (42.9%) of the CD20-negative DLBCL cases achieved a CR. In addition, the 3-year OS rate for CD20-negative DLBCL was only 35%, which was much less than CD20-positive DLBCL (74.1%) in our control group, and even the 5-year OS rate of conventional DLBCL (52%) before the rituximab era [34]. The reasons for the poor response and outcome to conventional chemotherapy in the current CD20-negative DLBCL series remain unclear. It is noteworthy that although the CD20-negative DLBCL included 5 cases with ALK-positive DLBCL, 2 cases with PBL, and 3 cases with DLBCL with plasmacytic differentiation, which are known for poor response and survival rates [8-14], only 2 cases with ALK-positive DLBCL died and 6 of the above-mentioned 10 DLBCL cases were alive at the time of last follow-up (the remaining 2 patients had no clinical data). We can thus assume that the poor response and clinical outcome of CD20-negative DLBCL is not entirely due to the inclusion of such specified subtypes. However, a high proportion of non-GCB types, a high proliferation index, and frequent extranodal involvement in CD20-negative DLBCL might be an explanation because these factors are associated with biological aggressiveness in conventional DLBCL [17,22,23]. Rituximab, in combination with chemotherapy, is the standard regimen for *de novo* CD20-positive DLBCL. However, rituximab may have little value in the initial therapy of *de novo* CD20-negative DLBCL patients. A lack of active targeted therapy for CD20-negative DLBCL might also contribute to the poor prognosis. Recent studies have indicated that a higher level of CD20 expression correlates with improved OS in B cell lymphoma patients treated with rituximab [35,36]. Furthermore, the study by Johnson et al. [37] demonstrated that DLBCL with reduced CD20 expression had a markedly inferior survival when treated with CHOP or rituximab-CHOP. Further investigations are warranted to evaluate the CD20-independent survival pathway and to develop new optimal therapy in CD20-negative DLBCL.

The IPI score is a useful tool in predicting outcome in typical DLBCL treated with conventional chemotherapy [34]. However, univariate analysis in our study failed to demonstrate an association between survival and IPI. In addition, except for a PS ≥ 2, extranodal involvement ≥ 2, and a SD/PD response to initial therapy, the other clinical characteristics (increased age, advanced stage, elevated LDH level, and bulky tumor) was not associated with inferior OS. Moreover, a recent report showed that an immunohistochemical biomarker, such as BCL-2, can predict OS in conventional DLBCL treated with CHOP [38]. Another study by Miller et al. [22] demonstrated that DLBCL patients with a high Ki-67 (≥ 80%) had a significantly worse outcome compared to those with a low Ki-67 (<80%). Similarly, we showed that a high Ki-67 (but not positive BCL-2) was associated with inferior survival in patients with CD20-positive DLBCL (data not shown). However, Ki-67 and BCL-2 did not have prognostic value in CD20-negative DLBCL. One possible reason for this negative result is that the biological behavior of CD20-negative DLBCL may be different from that of CD20-positive DLBCL.

## Conclusions

In conclusion, our study indicated that CD20-negavtive DLBCL is strikingly rare. With a higher proportion of non-GCB types, a higher proliferation index, and more frequent extranodal involvement compared to CD20-positive DLBCL, most of the CD20-negative DLBCL patients had a poor response and prognosis. Optimal treatment may require special consideration for these distinctive DLBCL cases. Further studies with a larger series are warranted to identify the clinicopathologic features of HIV-negative, CD20-negative DLBCL patients.

## Competing interests

The authors declare that they have no competing interests.

## Authors’ contributions

YJL and ZML performed the immunohistochemical staining, analyzed the results, and drafted the manuscript. WQJ conceived the study and coordinated the writing. ZML participated in the study design. HLR performed the pathologic analysis. YX, HQH, ZJX, SL, and WYL performed the case collection. All authors read and approved the final manuscript.
